# Association between dietary patterns and stroke in patients with type 2 diabetes mellitus in China: a propensity score-matched analysis

**DOI:** 10.1017/S1368980022000763

**Published:** 2022-08

**Authors:** Chenlu He, Wei Wang, Qian Chen, Ziyuan Shen, Enchun Pan, Zhongming Sun, Peian Lou, Xunbao Zhang

**Affiliations:** 1 School of Public Health, Xuzhou Medical University, 209 Tong Shan Road, Xuzhou, Jiangsu 221004, China; 2 Huai´an Center for Disease Control and Prevention, Huai´an, Jiangsu, China; 3 Xuzhou Center for Disease Control and Prevention, Xuzhou, Jiangsu, China

**Keywords:** Stroke, Type 2 diabetes, Dietary patterns, Latent class analysis, Propensity score matching

## Abstract

**Objective:**

This study aimed to examine the impact of different dietary patterns on stroke outcomes among type 2 diabetes mellitus (T2DM) patients in China.

**Design:**

Participants were enrolled by a stratified random cluster sampling method in the study. After collecting dietary data using a quantified FFQ, latent class analysis was used to identify dietary patterns, and propensity score matching was used to reduce confounding effects between different dietary patterns. Binary logistic regression and conditional logistic regression were used to analyse the relationship between dietary patterns and stroke in patients with T2DM.

**Setting:**

A cross-sectional survey available from December 2013 to January 2014.

**Participants:**

A total of 13 731 Chinese residents aged 18 years or over.

**Results:**

Two dietary patterns were identified: 61·2 % of T2DM patients were categorised in the high-fat dietary pattern while 38·8 % of patients were characterised by the balanced dietary pattern. Compared with the high-fat dietary pattern, the balanced dietary pattern was associated with reduced stroke risk (OR = 0·63, 95 %CI 0·52, 0·76, *P* < 0·001) after adjusting for confounding factors. The protective effect of the balanced model did not differ significantly (interaction *P* > 0·05).

**Conclusions:**

This study provides sufficient evidence to support the dietary intervention strategies to prevent stroke effectively. Maintaining a balanced dietary pattern, especially with moderate consumption of foods rich in quality protein and fresh vegetables in T2DM patients, might decrease the risk of stroke in China.

Over the past few decades, type 2 diabetes mellitus (T2DM) has become one of the highly prevalent and growing chronic diseases, severely threatening the Chinese population^(1)^. A series of epidemiological studies showed a positive and robust relationship between the risk of stroke and T2DM^(2,3)^. It is well known that diabetic patients with stroke have an extended hospital stay, poor prognosis and high mortality rate. The heavy burden of T2DM and stroke bringing to individuals and society worldwide is expected to increase in the next few years^(4)^. Common complications including hypertension, hypercoagulability, abnormal haemorheology, dyslipidaemia and other factors that occurred in T2DM patients may lead to arteriosclerosis. These factors interact with each other, resulting in a significantly increased risk of CVD^(5)^. Briefly, people with diabetes have a higher risk of stroke compared with non-diabetic patients^(6–8)^.

Stroke, as one of the most prominent complications of T2DM, is the frequent cause of death and long-term disability worldwide^(9)^. The age-standardised stroke mortality has declined globally over the past 20 years. However, the absolute number of stroke events, deaths and stroke burden increases substantially, especially in developing countries^(10,11)^. The number of stroke deaths in China accounts for about one-third of the global deaths of stroke, and the number of stroke patients has reached 13 million. China has become the country with the highest lifetime risk of stroke, with a massive burden of 39·3 %^(12)^. It is a remarkable fact that stroke is considered as a long-term and comprehensive disease caused by modifiable factors such as diet, physical activity, smoking and consumption of alcohol^(5,13,14)^.

Emerging evidence has revealed that dietary patterns also play an essential role in preventing stroke in the general population^(15–17)^. Substantial researches have been carried out over the past few decades to advance understanding of the impact of dietary patterns on stroke mechanisms^(15,16,18)^. Adherence to Dietary Approaches to Stop Hypertension (DASH) and prudent dietary pattern demonstrated a protective effect on stroke, whereas inverse associations were observed in the Western dietary pattern^(18–20)^. The Mediterranean diet was associated with increased CVD stroke^(21–23)^. The dietary patterns were not consistent across global regions, such as Europe and Asia, due to the complexity and diversity of food^(15,24)^. A prospective cohort study of 13 055 adults in China suggested a negative association between traditional dietary pattern, which emphasises a high intake of rice, pork, fish, poultry and fresh vegetable but low intake of wheat and CVD. On the contrary, modern dietary pattern characterised by a high intake of fruit, soya milk and fast food was associated with an increased risk of CVD^(16)^.

Although multiple epidemiological studies have extensively focused on dietary patterns and their effect on stroke in a general population^(16,25,26)^, little was known regarding the relationship between dietary patterns and stroke in patients with T2DM. For this purpose, our study was thus far the first study aimed to explore the impact of different dietary patterns on stroke outcomes in T2DM patients in China.

## Materials and methods

### Participants and procedure

This was a cross-sectional study conducted among T2DM patients aged ≥ 18 years in China from December 2013 to January 2014. A representative sample including forty-four towns/streets selected from sixty-five towns/streets of two regions in the south and north of Jiangsu Province randomly was obtained using a stratified cluster random sampling method. Resident health records of 20 053 T2DM patients were enrolled in the essential public health service management system. Three groups of participants were excluded: (1) participants diagnosed with stroke before T2DM, (2) patients with a duration of T2DM < 1 year and (3) participants with missing values of questionnaires. Finally, 13 731 participants diagnosed with T2DM patients between 1982 and 2012 were successfully involved in the analysis.

### Data collection

Information regarding demographic characteristics (age, gender, region, residence, level of education, marital status, household income, employment, duration of diabetes), health-related behaviours (smoking, drinking and physical activity) and related diseases (hypertension, dyslipidaemia, overweight and obesity) was collected through standard paper questionnaires by well-trained staff members.

### Definition of variables

Fasting plasma glucose (FPG) ≥ 7·0 mmol/l on at least two separate occasions by previous diagnosis of professionals or use of medication was defined as T2DM^(27)^. Stroke was defined as sudden onset of non-convulsive and focal neurological deficit lasting more than 24 h. It was classified as ischaemic stroke, subarachnoid haemorrhage, brain haemorrhage and other types of stroke by previous physician diagnosis. Self-reported stroke patients were defined as those who answered ‘NO’ to this question ‘Have you ever been diagnosed with stroke by a neurologist?’ People who have smoked at least 100 cigarettes in one’s lifetime was defined as a smoker. Drinking more than once a month was considered drinking. Physical activity information was obtained through the global physical questionnaire^(28)^. The level of physical activity was calculated using the metabolic equivalent value of various physical activities and the duration and frequency of multiple activities. For each physical activity group, participants reporting no physical activity were first classified as zero physical activity, with the remainder divided into tertiles of the level of physical activity: low level (first tertile), moderate level (second tertile) and high level (third tertile). BMI was calculated by weight in kilograms divided by the square of height in metres. It was divided into four levels: underweight (BMI < 18·5), normal weight (18·5 ≤ BMI < 24·0), overweight (24·0 ≤ BMI < 28·0) and obesity (BMI ≥ 28·0)^(29)^. Previous studies have confirmed that these covariates selected in this study are related to dietary patterns and stroke in propensity score matching (PSM)^(11,30–33)^.

### Dietary intake assessment

Dietary intake was evaluated through a validated seventeen FFQ by well-trained investigators from December 2013 to January 2014. Data on intake and frequency of various foods consumed in the last year were collected using the paper questionnaire. Information on average daily intake of each kind of food which included rice and noodles, whole grains, poultry meat, livestock meat, aquatic products, fresh vegetables, fresh fruits, soyabean products, eggs, dairy products, nuts, fried foods, cakes, salted products, juice, beverages and carbonated drinks was calculated (g/d).

### Propensity score matching

Patients were matched based on their propensity scores to dietary patterns. The propensity scores of each participant were calculated by propensity model (multivariate logistic regression model) founded on fifteen confounding factors including demographic characteristics (age, duration of diabetes, gender, residence, household income, level of education, marital status and occupation), health-related behaviours (smoke, drink and effective exercise), related diseases (hypertension, dyslipidaemia, overweight or obesity) and HbA1c levels.

Each participant in the high-fat pattern group was matched to an object in the balanced pattern group through nearest neighbour matching in a 1:1 ratio with a caliper width of 0·02. The participants after matching were used for subsequent analyses. To evaluate matching effect of the sample, we acquired the absolute standardised differences based on fifteen confounding factors before and after PSM.

### Statistical analysis

Statistical analyses were performed on software Mplus version 7.0 and R version 4.0.2. The descriptive statistics including percentage and median were used to describe the demographic characteristics. The chi-squared test and the Mann–Whitney *U* test were used to assess the demographic characteristics differences between dietary patterns before and after PSM. Foods were first aggregated into seventeen mutually exclusive foods to determine dietary patterns using latent class analysis. For each kind of food, participants were first classified as those reporting zero intake, with the remainder divided into tertiles of intake and then categorised into three categories: low intake (first tertile), moderate intake (second tertile) and high intake (third tertile). The best-fitting model was determined on the basis of information criteria-based metrics, including the Akaike information criterion (AIC); Bayesian information criterion (BIC); sample-size-adjusted Bayesian information criterion (aBIC); Lo–Mendell–Rubin likelihood ratio test (LMR); Bootstrap likelihood ratio test (BLRT); entropy and substantive interpretation. Different dietary patterns were described based on the conditional probabilities of the corresponding foods.

Binary logistic regression was used for the entire population, and conditional logistic regression was used for propensity score-matched population in subgroup analysis to assess the risk of stroke and OR, and related 95 % CI were calculated. *P* < 0·05 (bilateral) was considered statistically significant.

## Results

### Demographic characteristics of type 2 diabetes mellitus patients

A total of 867 (6·3 %) patients had a stroke. The respondents’ ages ranged from 21 to 94 years with a mean age of 63 years. Close to two-thirds of participants (61·0 %) were 60 years and above. Of them, a more significant proportion (60·7 %) of them were females. The highest proportion of participants was residing in urban–rural areas (53·9 %). Further, nearly three-fourth (55·0 %) received junior school or below. With regard to health-related behaviours, there were 27·3 % current smokers and 16·8 % drinkers. In terms of the related diseases, the largest proportion of patients (63·2 %) was overweight or obesity, followed by hypertension (56·3 %), and the least proportion was dyslipidaemia (16·8 %).

### Latent class analysis

Model fitting statistics for one to six latent classes were performed in the latent class analysis models among patients with T2DM. Along with the number of estimated classes increased, the Akaike information criterion, Bayesian information criterion and sample-size-adjusted Bayesian information criterion indicated monotonically improving fit. On the other hand, entropy remained consistently above 0·60 and increased with the number of categories. NYLUND *et al*. found that the Lo–Mendell–Rubin test was the most sensitive index to the classification of potential categories in the Monte Carlo simulation study. When non-significant (*P* > 0·05), the Lo–Mendell–Rubin and the Bootstrap likelihood ratio test demonstrated that the two-class model fits the data better than the three-class model significantly^(34)^. Founded on model fit tests and the aim of parsimony, the two-class model was considered as the best solution of latent dietary patterns (Table 1).

**Table 1:** LCA fit indices of dietary patterns among patients

Model	AIC	BIC	aBIC	Entropy	LMR	BLRT	Class probability
1	469 591	469 983	469 817	–	–	–	–
2	457 755	458 545	458 211	0·673	<0·001	<0·001	0·612/0·388
3	450 542	451 732	451 230	0·685	0·818	<0·001	0·350/0·267/0·383
4	447 070	448 659	447 988	0·687	0·760	<0·001	0·253/0·308/0·236/0·202
5	444 202	446 189	445 350	0·698	0·842	<0·001	0·240/0·225/0·144/0·196/0·195

LCA, latent class analysis; AIC, Akaike information criterion; BIC, Bayesian information criterion; LMR, Lo–Mendell–Rubin; BLRT, Bootstrap likelihood ratio test.

**Fig. 1:**
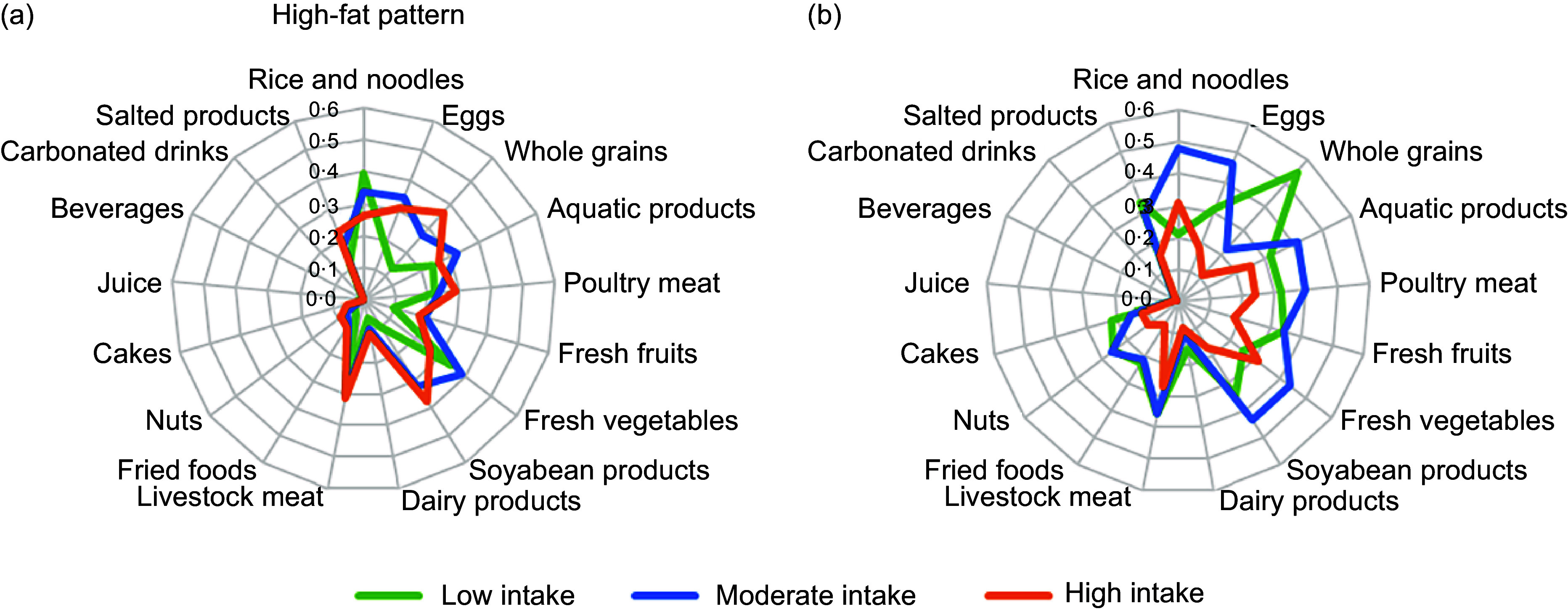
Probabilities of consumption for selected food items by dietary patterns derived from latent class analysis. The green line: low intake of food; the blue line: moderate intake of food; the red line: high intake of food

Of the 13 731 subjects aged 18 years or over in China, 61·2 % were classified as consuming the high-fat pattern, while 38·8 % were described as the balanced pattern. Class 1 labelled the high-fat pattern and showed a higher probability of moderate to high consumption of whole grains, poultry meat, livestock meat, aquatic products, soyabean products, eggs and fresh vegetables, and low consumption of rice and noodles. There is no consumption of fresh fruits, dairy products, fried foods, nuts, cakes, juice, beverages, carbonated drinks and salted products in the high-fat pattern. Subjects in class 2, the balanced pattern, reported a higher probability to consume moderate consumption of rice and noodles, livestock meat, aquatic products, eggs, fresh vegetables, soyabean products and low consumption of whole grains, poultry meat, fresh fruits and salted products. There is no consumption of dairy products, fried foods, nuts, cakes, juice, beverages and carbonated drinks in the balanced pattern (Fig. 1).

### Characteristics of dietary patterns before and after propensity score matching

Based on the different dietary patterns before matching, participants were divided into high-fat (*n* 8401(61·2 %)) and balanced (*n* 5330 (38·8 %)) patterns. There were statistically significant differences between two patterns in all covariates except for age, blood glucose control, smoking and drinking status (Table 2). Overall, 867 (6·3 %) patients had a stroke. Notably, balanced pattern patients were the best possibility to be women and overweight or obese.

**Table 2:** The comparisons of characteristics by LCA for patients with T2DM before and after matching

Characteristic	Before PSM (*N* 13 731)					After PSM (*N* 9970)				
	Balanced pattern (*N* 5330)		High-fat pattern (*N* 8401)		*P*	Balanced pattern (*N* 4985)		High-fat pattern (*N* 4985)		*P*
	*N*	%	*N*	%		*N*	%	*N*	%	
Age at diagnosis (years)					0·935					0·237
Median	63		63			56		56		
Interquartile range	57, 69		56, 69			50, 63		49, 63		
Duration of diabetes (years)					<0·001					0·214
Median	5		5			5		5		
Interquartile range	3, 10		2, 9			3, 10		3, 10		
HbA1c (%)					0·424					0·304
≥7	3154	59·2	5027	59·8		2955	59·3	2982	59·8	
<7	2164	40·6	3352	39·9		2030	40·7	2003	40·2	
Gender					0·021					0·207
Male	2032	38·1	3369	40·1		1905	38·2	1844	37·0	
Female	3298	61·9	5032	59·9		3080	61·8	3141	63·0	
Residence					<0·001					<0·001
Urban	2918	54·7	3412	40·6		2747	55·1	3054	61·3	
Rural	2412	45·3	4989	59·4		2238	44·9	1931	38·7	
Marital status					<0·001					0·190
Married/cohabiting	606	11·4	1142	13·6		571	11·5	530	10·6	
Other	4710	88·4	7211	85·8		4414	88·5	4455	89·4	
Education level					0·078					0·500
Junior school or below	3717	69·7	5961	80·0		3484	69·9	3453	69·3	
Other	1606	30·1	2407	28·7		1501	30·1	1532	30·7	
Employment					0·011					0·044
Physical activity	1866	35·0	3117	37·1		1738	34·9	1643	33·0	
Other	3457	64·9	5265	62·7		3247	65·1	3342	67·0	
Household income (yuan)					<0·001					0·673
≤150 000	5069	95·1	8082	96·2		4120	82·6	4104	82·3	
>150 000	240	4·5	277	3·3		865	17·4	881	17·7	
Smoking					0·071					0·964
Yes	1410	26·5	2333	27·8		1325	26·6	1323	26·5	
No	3898	73·1	6006	71·5		3660	72·4	3662	73·5	
Drinking					0·237					0·460
Yes	924	17·3	1387	16·5		858	17·2	886	17·8	
No	4401	82·6	6981	83·1		4127	82·8	4099	82·2	
Exercise (MTES-hours/d)	8·0	3·4,15·4	7·1	2·7,14·9	0·002	8·0	3·4–15·0	7·4	2·7,16·0	0·016
Hypertension					<0·001					0·006
Yes	3191	59·9	4533	54·0		3006	60·3	3139	63·0	
No	2102	39·4	3693	44·0		1979	39·7	1846	37·0	
Dyslipidaemia					<0·001					0·540
Yes	893	16·8	1408	16·8		840	16·9	863	17·3	
No	4437	83·2	6993	83·2		4145	83·1	4122	82·7	
Overweight or obesity					0·035					0·343
Yes	3312	62·1	5372	63·9		3113	62·4	3067	61·5	
No	2009	37·7	3018	35·9		1872	27·6	1918	38·5	

LCA, latent class analysis; PSM, propensity score matching; T2DM, type 2 diabetes mellitus.

**Fig. 2:**
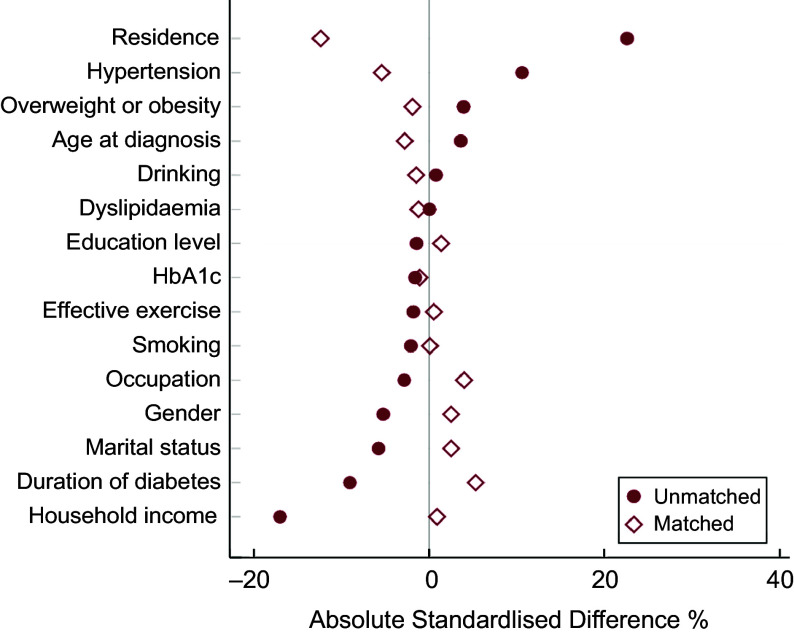
Absolute standardised differences in fifteen covariates between high-fat pattern and balanced pattern patients before and after matching. The maroon solid circle represents absolute standardised differences before matching, and the maroon hollow diamond represents absolute standardised differences after matching

A total of 4985 balanced pattern patients could be propensity-matched to high-fat pattern patients, creating a new sample of 9970 patients (4985 balanced pattern patients, 4985 high-fat pattern patients). After matching, the balance between two dietary patterns remarkably improved, as none of the confounding factors was significantly different except for occupation, physical activity level, hypertension and residence (Table 2). The groups after PSM were also well balanced in other variables not included in the model. The absolute standardised differences were < 10 % for all covariates between different dietary patterns except for residence (Fig. 2).

### Association between different dietary patterns and stroke in type 2 diabetes mellitus patients

As illustrated in Table 3, there were 583 (5·8%) events of stroke after PSM. Conditional logistic regression models exhibited that the balanced pattern was correlated with a statistically significant decrease in the risk of stroke (OR = 0·61; 95 %CI 0·51, 0·72; *P* < 0·001). To ensure that these results were not confused by the covariates with unsatisfactory matching, we evaluated the risk of adverse outcomes after adjusting for unbalanced factors after PSM, including occupation, physical activity level, hypertension and residence. After adjustment, the balanced pattern was also to be correlated with decreased risk of stroke (adjusted OR = 0·63; 95 % CI 0·52, 0·76; *P* < 0·001). The results of the propensity-matched analysis were authenticated in the whole population (OR = 0·61; 95% CI 0·52, 0·71; *P* < 0·001) (Table 3).

**Table 3:** Prevalence of stroke and impact of different dietary patterns on stroke outcome in the entire and PS-matched population

Events (stroke)	Prevalence						Unadjusted risk			Adjusted risk*		
	Total		Balanced pattern		High-fat pattern				*P*			*P*
	OR	95 % CI	OR	95 % CI	OR	95 % CI	OR	95 % CI		OR	95 % CI	
Entire population	867	6·3	246	4·6	621	7·4	0·61	0·52–0·71	<0·001	0·59	0·50–0·69	<0·001
PS-matched population	583	5·8	220	4·4	363	7·3	0·61	0·51–0·72	<0·001	0·63	0·52–0·76	<0·001

(Balanced pattern *v*. high-fat pattern); PS, propensity score; PSM, propensity score matching.

Data are expressed as *n* (%) where appropriate. OR and CI were derived from logistic regression models.

Binary logistic As per journal style, unlinked footnotes are not allowed. Hence, please link them with symbols in the sequential order.regression was used for the entire population, and conditional logistic regression was used for PS-matched population.

* In the entire population analyses, adjustment was for the PS; in the propensity-matched population analysis, adjustment was for unbalanced factors after PSM.

### Subgroup analysis

**Fig. 3:**
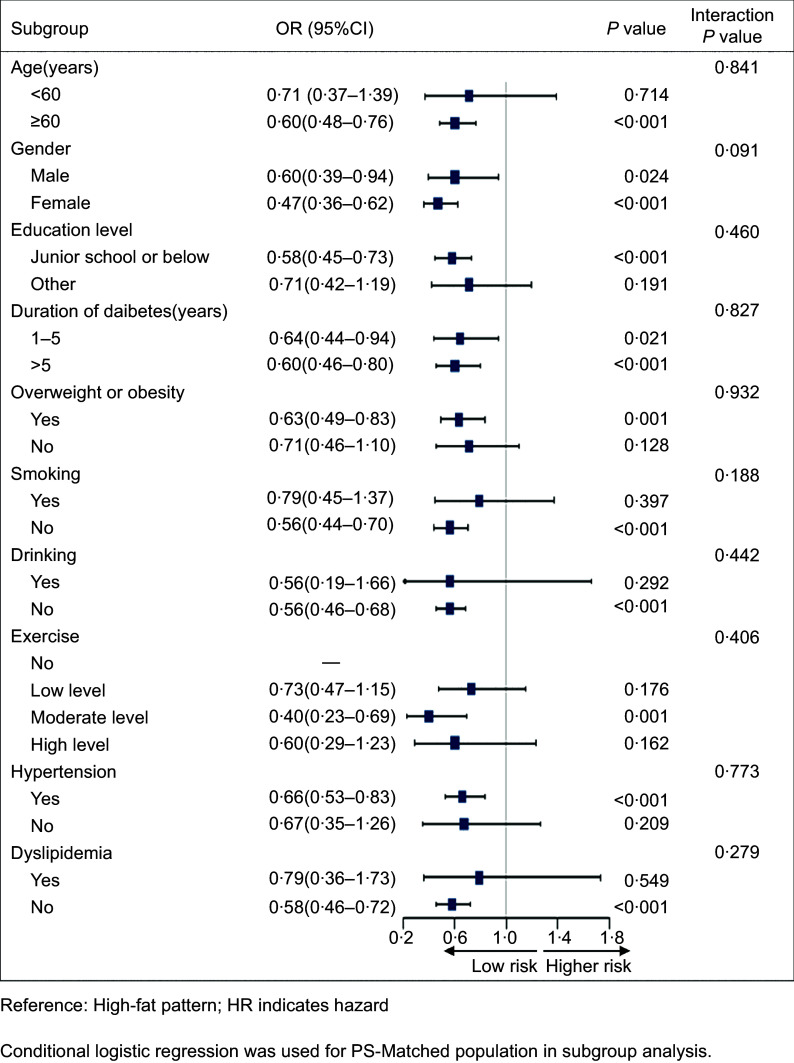
Impact of different dietary patterns on stroke outcomes by subgroups after propensity matching

To confirm the association between two different dietary patterns and stroke in various subgroups, we carried out subgroup analyses using conditional logistic regression models. The OR for balanced pattern were constantly < 1·0 in all subgroups, demonstrating the decreased risk of stroke. However, the protective effect of the balanced model did not differ significantly (interaction *P* > 0·05) (Fig. 3).

## Discussion

To our knowledge, our study is the first study to focus on the impact of different dietary patterns on stroke outcomes in T2DM patients. Compared with traditional methods such as factor analysis to assess dietary patterns, latent class analysis has its unique superiority. First, it has less restrictive premises and is very flexible. Second, it is suitable for clustering analysis of both hierarchical variables and categorical variables. In addition, latent class analysis uses probability to classify based on a specific probability model, which is less arbitrary and more in line with the objective reality. The results showed that there were differences in dietary patterns among patients with T2DM. The typical dietary characteristics of two dietary patterns in T2DM patients contained a variety of foods rich in quality protein such as soyabean products or whole grains and fewer intake of fruits and rice, which may be due to the transition to a low glycaemic index diet after diabetes considering their state of health, especially the reduction in the intake of foods that are likely to contribute to poor control of glycaemia^(35,36)^.

Participants exhibiting adherence to the balanced pattern consumed moderate amounts of beneficial foods such as white meat, eggs, soyabean products and fresh vegetables tended to a Mediterranean diet, which was beneficial to stroke prevention^(21–23,37)^. A healthy dietary pattern could also affect other modifiable risk factors, namely obesity, diabetes and dyslipidaemia, thereby influencing the risk of stroke^(38)^. The association between high-fat pattern and increased stroke risk might be mediated through obesity or central obesity. Patients in the high-fat pattern consumed a high intake of staple foods, including rice and whole grains rich in starch and high-fat foods. A series of researches investigated the relationship between meat consumption and the stroke risk and exhibited that consumption of red meat, which was a source of SFA, was related to an increased risk of stroke^(39–41)^, which was in agreement with our findings.

However, the results of a series of studies regarding red meat were also controversial^(41–43)^. Several studies found that excessive consumption of red meat was correlated with an increased stroke risk, while moderate consumption of red meat could not result in lipid structural changes or significant blood pressure increases, which supported our conclusion^(41–43)^. On the other hand, there is little literature on revealing that high meat intake was negatively correlated with CHD and stroke. High-fat intake, including SFA and unsaturated fatty acid, was associated with reduced stroke risk, and no adverse effects of fat intake on CVD were observed^(44,45)^. In contrast, consumption of white meat had a protective impact on stroke^(40,46)^. Based on evidence mentioned above, further researches are needed on the association between red meat and stroke in patients with T2DM.

Participants in the balanced pattern also consumed high levels of soyabean products, which may have a protective effect on stroke^(47,48)^. Beans are rich in protein, carbohydrates, fibre and various micronutrients (such as phytochemicals)^(49)^. A series of meta-analyses of prospective cohort researches on the relationship between eggs and stroke have provided conflicting findings^(41,49–52)^. Eggs are a primary source of dietary cholesterol considering the weak correlation between dietary cholesterol and blood cholesterol, and they are also an affordable and common source of high-quality protein, unsaturated fatty acids, Fe and phospholipids^(53,54)^. Also, eggs were demonstrated to have a protective effect on stroke, which are consistent with the results of our study^(41,49,52,55)^.

Furthermore, it is necessary to underline moderate to high consumption of fresh fruits and vegetables. Several epidemiological studies have ascertained an inverse correlation between high consumption of fruits and vegetables and stroke risk^(56–58)^. Fruits and vegetables contain a variety of nutrients and phytochemicals, including fibre, vitamin C, carotenoids, antioxidants, K and flavonoids, which might decrease the risk of stroke through a combination of biological mechanisms^(47,49,58,59)^. Also, a high intake of fruits and vegetables can indirectly decrease the risk of chronic diseases by replacing unhealthy foods high in saturated fat, blood transfusions, glycaemic load and Na. Chinese populations tended to eat vegetables that have been cooked with excessive salt resulting in an increased risk of stroke.

Notably, adherence to a long-term vegetarian dietary pattern is most probably to lead to a decline in immunity, due to the lack of digestible animal protein and fat, resulting in large fluctuations in blood glucose^(60,61)^. Dietary control in patients with diabetes should not be simply considered that low intake and single intake of food were beneficial to participants. Therefore, improving the nutritional status of malnutrition should be encouraged as part of important prevention for stroke patients.

There are some limitations to the study. First, the study sample was selected from Jiangsu province and not representative of the total T2DM patients in China. Thus, this conclusion cannot be generalised to China or even the whole world. Second, this study was not a cohort design; consequently, we could only suggest correlations. We had no access to get reliable inference of causality between dietary patterns and stroke. Thus, further cohort studies are needed in the future to confirm our results. However, we had no access to reveal more robust reliable inferences of causality. Further prospective cohort studies are needed to verify our findings in the future. Third, although some potential confounding factors have been adjusted, the retrospective method of food frequency survey and self-reporting history of diseases cannot avoid the possibility of recall bias and other unknown confounding factors, which may affect the results of the analysis. Fourth, subgroup analysis may destroy the results after PSM, resulting in bias, which makes the conclusions less robust. The results of subgroup analysis can only be used as exploratory analysis to provide clues for studies, and further studies are needed for confirmation in the future.

## Conclusions

This study provides sufficient evidence to support the dietary intervention strategies to prevent stroke effectively. In our study, we evaluate whether a particular dietary pattern was correlated with stroke risk in patients with T2DM, adjusting for other known risk factors. Our findings indicated that maintaining a balanced dietary pattern, especially with moderate consumption of high-quality protein food and a high intake of vegetables instead of low consumption of various foods merely, was correlated with a decreased risk of stroke in T2DM patients in China.
